# Arthrosis diagnosis and treatment recommendations in clinical practice: an exploratory investigation with the generative AI model GPT-4

**DOI:** 10.1186/s10195-023-00740-4

**Published:** 2023-11-28

**Authors:** Stefano Pagano, Sabrina Holzapfel, Tobias Kappenschneider, Matthias Meyer, Günther Maderbacher, Joachim Grifka, Dominik Emanuel Holzapfel

**Affiliations:** 1 Department of Orthopaedic Surgery, University of Regensburg, Asklepios Klinikum, Bad Abbach, Germany; 2 Department of Neonatology, University Children’s Hospital Regensburg, Hospital St. Hedwig of the Order of St. John, University of Regensburg, Regensburg, Germany

**Keywords:** Artificial intelligence, ChatGPT-4, Large language model, Orthopaedics, Total joint replacement, Arthrosis

## Abstract

**Background:**

The spread of artificial intelligence (AI) has led to transformative advancements in diverse sectors, including healthcare. Specifically, generative writing systems have shown potential in various applications, but their effectiveness in clinical settings has been barely investigated. In this context, we evaluated the proficiency of ChatGPT-4 in diagnosing gonarthrosis and coxarthrosis and recommending appropriate treatments compared with orthopaedic specialists.

**Methods:**

A retrospective review was conducted using anonymized medical records of 100 patients previously diagnosed with either knee or hip arthrosis. ChatGPT-4 was employed to analyse these historical records, formulating both a diagnosis and potential treatment suggestions. Subsequently, a comparative analysis was conducted to assess the concordance between the AI’s conclusions and the original clinical decisions made by the physicians.

**Results:**

In diagnostic evaluations, ChatGPT-4 consistently aligned with the conclusions previously drawn by physicians. In terms of treatment recommendations, there was an 83% agreement between the AI and orthopaedic specialists. The therapeutic concordance was verified by the calculation of a Cohen’s Kappa coefficient of 0.580 (*p* < 0.001). This indicates a moderate-to-good level of agreement. In recommendations pertaining to surgical treatment, the AI demonstrated a sensitivity and specificity of 78% and 80%, respectively. Multivariable logistic regression demonstrated that the variables reduced quality of life (OR 49.97, *p* < 0.001) and start-up pain (OR 12.54, *p* = 0.028) have an influence on ChatGPT-4’s recommendation for a surgery.

**Conclusion:**

This study emphasises ChatGPT-4’s notable potential in diagnosing conditions such as gonarthrosis and coxarthrosis and in aligning its treatment recommendations with those of orthopaedic specialists. However, it is crucial to acknowledge that AI tools such as ChatGPT-4 are not meant to replace the nuanced expertise and clinical judgment of seasoned orthopaedic surgeons, particularly in complex decision-making scenarios regarding treatment indications. Due to the exploratory nature of the study, further research with larger patient populations and more complex diagnoses is necessary to validate the findings and explore the broader potential of AI in healthcare.

*Level of Evidence*: Level III evidence.

**Supplementary Information:**

The online version contains supplementary material available at 10.1186/s10195-023-00740-4.

## Introduction

In an era characterized by rapid technological development, the global community stands at a turning point. The rise of artificial intelligence (AI), captured by its extensive applications, presents both boundless opportunities and inherent challenges. Among the technological vanguards, generative writing systems, such as GPT-4 by OpenAI (San Francisco, USA) – commonly known as ChatGPT – have emerged as paragons of this evolution. Launched on 14 March 2023, this AI model boasts an expansive database updated until September 2021 and exhibits proficiency in assimilating both text and other data inputs to produce textual outputs. Embedded in the transformer architecture with an imposing 170 trillion nodes, GPT-4 excels in predicting subsequent tokens in a sequence, mirroring human competence across diverse professional and academic settings [1]. Ongoing research from Eloundou et al. indicates that large language models (LLMs) such as GPT-4 might affect more than 80% of the US workforce, altering more than half of tasks currently undertaken by workers in about 19% of analysed scenarios [2].

In the specific field of healthcare, significant changes are also expected with repercussions on the entire global health system [3–5]. Recent literature underscores the multifaceted advantages of ChatGPT, emphasizing its potential in refining scientific literature, health research, clinical practice, and medical education. Concurrently, concerns permeate regarding ethical considerations, legal ramifications, transparency, plagiarism, inaccuracies, and cybersecurity vulnerabilities [6]. These challenges, although substantial, do not negate the impressive capabilities demonstrated by this technology.

The integration of AI, particularly machine learning (ML), with electronic medical record systems has led to transformative advancements in orthopaedics. ML’s adeptness in handling big datasets has facilitated tasks such as fracture identification from radiographs and osteoarthritis staging through gait analysis [7, 8].

Thanks to its abilities, ChatGTP has shown immense reliability in performing tests and specialized exams. Remarkably, this AI-based writing system managed to pass both the German medical state examination and the United States Medical Licensing Examination without any issues [9, 10].

GPT-4 also offers different potential applications in arthroplasty, ranging from enhancing diagnosis and treatment plans to optimizing preoperative planning, supporting intraoperative procedures, and guiding postoperative rehabilitation [11].

Given our expertise in orthopaedics, we believe osteoarthritis – a prevalent condition – serves as a suitable testbed for such an examination [12]. With this background, we postulate the hypothesis that, while AI like GPT-4 possesses outstanding diagnostic abilities, it may not yet match the nuanced expertise of a senior orthopaedic surgeon specialized in joint replacement at a high-volume university hospital when determining indications for total joint arthroplasty (TJA).

## Materials and methods

### Study design and objectives

A retrospective observational study with an explorative character was undertaken at our hip and knee prosthetics outpatient clinic. The primary aim was to assess ChatGPT-4’s (6 July 2023 Version, OpenAI, San Francisco, USA) proficiency in orthopaedic diagnostics, in particular on its accuracy in diagnosing gonarthrosis or coxarthrosis and its therapeutic recommendations aligned with actual clinical decisions.

### Patient selection and data extraction

We started a comprehensive review of medical records from patients presenting with hip or knee disorders at our outpatient clinic between 2022 and 2023. From the patient data available, we intentionally selected a study sample of 100 adult patients, ensuring an equal distribution of 50 patients with knee disorders and 50 with hip disorders. This target was also chosen to achieve an adequate population size due to the exploratory nature of the study. Furthermore, within these two groups, we maintained an even distribution in terms of treatment recommendation, with 25 patients advised for conservative treatments and 25 for surgical interventions in each group. Additionally, our sample also ensured gender balance, with 50 male and 50 female participants.

To qualify for the study, the adult patients needed to exhibit clear clinical signs of either gonarthrosis or coxarthrosis. Their medical history had to be comprehensive, including symptomatology, outcomes of physical examinations, radiographic interpretations provided by a certified radiologist, and treatment recommendations made by an orthopaedic specialist.

The outpatient medical record had to include following data:

- *Demographics:* age, sex.
- *Clinical diagnosis:* presence of gonarthrosis or coxarthrosis.
- *Anatomical details:* affected joint and side.
- *Comorbidities:* general systemic conditions, specific comorbidities such as lumbago, rheumatic diseases, obesity, prior arthroplasty on the opposite side and record of any prior surgery related to the joint.
- *Clinical history:* duration of symptoms, various pain symptoms, need for pain medication, previous therapy such as intraarticular injections and physiotherapy.
- *Physical examination:* clinical inspection results, joint mobility assessments and other diagnostic signs.
- *Radiological findings:* radiological report of the X-ray, and if available, magnetic resonance imaging (MRI) findings of the considered joint.
- *Treatment suggestion:* recommendation of the orthopaedic specialist regarding conservative or operative therapy consisting in a total joint arthroplasty.

Medical records that seemed inconsistent, especially those devoid of the essential symptom descriptions, physical examination findings, or radiographic readings, were dismissed.

**Fig. 1:**
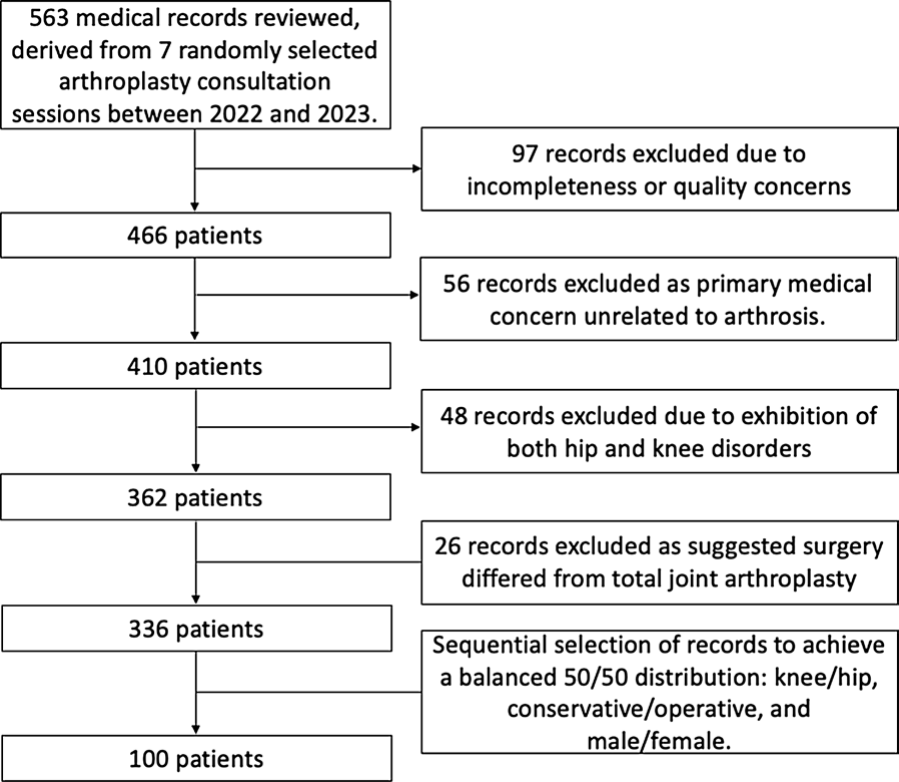
Flowchart illustrating the patient selection process for the study

Those for whom the primary medical concern was not related to arthrosis, or who presented with disorders in hip and knee joint simultaneously, were also omitted from the study. Moreover, patients advised for surgery were excluded from the study if the procedure indicated was not specifically a total hip or knee arthroplasty (Fig. 1).

### Data input into ChatGPT-4

Given the sensitive nature of medical data, a meticulous anonymization process was followed before any clinical record was entered into ChatGPT-4. Additionally, all direct or explicit references to arthrosis as well as the treatment recommendations were removed from the medical report. Although the radiologic images were graded for osteoarthritis using the Kellgren and Lawrence (KL) classification system [13] to characterize our patient cohort, this specific classification was not included in the analysis. Instead, only a descriptive radiological report was used, from which all direct references to arthrosis were removed before input into ChatGPT-4. To maintain the integrity of the study and to ensure that ChatGPT-4 was making independent assessments, each record was entered into a fresh input page, preventing any possible influence from previous data.

ChatGPT-4 was provided with comprehensive anonymised patient information, which included descriptions of symptoms, results of physical examinations and radiographic interpretations with the expectation to generate a differential diagnosis, to rank possible disorders based on likelihood and to suggest relevant therapeutic recommendations. The specifics of this task can be found in Additional file 1 under ‘Clinical Query Input to ChatGPT-4—Case Prompt’.

In the responses generated by ChatGPT-4, the generative model gives a series of potential diagnoses and corresponding treatment options, each accompanied by a specific percentage. These percentages signify the model’s calculated confidence in the likelihood of each diagnosis or the appropriateness of each treatment option on the basis of its learning algorithms and the medical data it has been trained on. Essentially, the percentages reflect how closely the input data – symptoms, physical examination results, and radiographic interpretations – match the information within the model’s training datasets, indicating the probability that a particular diagnosis is correct or a specific treatment is suitable given the patient’s unique presentation.

For statistical analysis, a standardized approach was then employed to interpret ChatGPT-4’s responses. From the list of diagnoses and therapeutic strategies the model provided, we selected as the model’s primary suggestion the single option in each category with a confidence score surpassing 50%. This threshold was chosen to prioritize options with a higher level of algorithmic certainty. In analysing ChatGPT-4’s responses, we noted that the model often suggested multiple conservative treatments. To accommodate this in our statistical analysis, we combined the confidence scores of different treatments within the same category (conservative or surgical). For instance, if physical therapy had a 30% confidence score and pain medication 25%, we would aggregate them, achieving a cumulative 55% confidence for conservative treatment. However, for surgical recommendations, we only accounted for total arthroplasty suggestions. The primary category surpassing a 50% total confidence was then taken as ChatGPT-4’s main recommendation.

To offer a clearer understanding of ChatGPT-4’s response, a chosen example from its feedback is described in Additional file 1 under ‘Case-Based ChatGPT-4 Response’.

### Statistical analysis

Continuous variables were presented as mean (standard deviation, SD), while categorical data were expressed using absolute (*n*) and percentage (percentage) frequencies. For comparing continuous data, two-sided t-tests were employed, and categorical data comparisons were executed using the chi-squared test.

The accuracy of ChatGPT-4 in deducing disorders and giving recommendations on the basis of the provided records was assessed using sensitivity, specificity, positive predictive value (PPV) and negative predictive value (NPV), considering the physician’s therapeutic advice as the gold standard.

To assess the interrater reliability between ChatGPT-4’s therapeutic advice and the physicians’ recommendations, Cohen’s Kappa statistic was used [14].

A multivariable logistic regression was then employed to test any associations between ChatGPT-4’s therapeutic suggestions and parameters such as patient demographics, medical backgrounds and outcomes from clinical examinations, taking care to account for confounders.

Given the exploratory nature of this study, a power calculation was not performed.

Patient data were collected using the in-house database (ORBIS, Agfa healthcare). IBM SPSS Statistics version 29 was the software of choice for all statistical analyses, and the significance level was set at a two-sided *p* ≤ 0.050.

The study was conducted upon approval from the Ethics Committee of the University of Regensburg (protocol no. 23-3404-104‬).‬‬

## Results

### Demographic and clinical characteristics

Our cohort consisted of 100 patients, with an equal distribution between knee (*n* = 50) and hip (*n* = 50) arthrosis. Both subpopulations were balanced in terms of gender and treatment approach: 25 male patients and 25 female patients, with 25 suggested for conservative treatment and 25 for surgical intervention in each group.

The study participants presented consistent attributes across age, clinical diagnosis and comorbidities. The only significant disparities were observed in the affected side (*p* = 0.012) and preceding interventions within the knee group (*p* < 0.001). Comprehensive data can be found in Table 1.

**Table 1:** Comparative demographics, clinical characteristics and comorbidities across knee and hip disorder groups

	Total (*n* = 100)	Knee (*n* = 50)	Hip (*n* = 50)	*p*-value
Age (years)				0.943
Range	35–88	35–88	41–86	
Mean (± SD)	64.7 (± 11.2)	64.7 (± 11.1)	64.6 (± 11.2)	
Sex				1.00
Female	50 (50.0%)	25 (50.0%)	25 (50.0%)	
Male	50 (50.0%)	25 (50.0%)	25 (50.0%)	
Systemic diseases				0.840
Yes	57 (57.0%)	28 (56.0%)	29 (58.0%)	
No	43 (43.0%)	22 (44.0%)	21 (42.0%)	
Side				0.012*
Right	44 (44.0%)	22 (44.0%)	22 (44.0%)	
Left	33 (33.0%)	11 (22.0%)	22 (44.0%)	
Both sides	23 (23.0%)	17 (34.0%)	6 (12.0%)	
Back pain				0.401
Yes	15 (15.0%)	6 (12.0%)	9 (18.0%)	
No	85 (85.0%)	44 (88.0%)	41 (82.0%)	
Rheumatic diseases				0.081
Yes	9 (9.0%)	7 (14.0%)	2 (4.0%)	
No	91 (91.0%)	43 (86.0%)	48 (96.0%)	
Obesity**				0.114
Yes	23 (23.0%)	15 (30.0%)	8 (16.0%)	
No	45 (45.0%)	20 (40.0%)	25 (50.0%)	
Prior surgery				< 0.001*
Yes	14 (14.0%)	13 (26.0%)	1 (2.0%)	
No	86 (86.0%)	37 (74.0%)	49 (98.0%)	
Prosthesis opposite side				0.603
Yes	18 (18.0%)	8 (16.0%)	10 (20.0%)	
No	82 (82.0%)	42 (84.0%)	40 (80.0%)	
Treatment				1.00
Conservative	50 (50.0%)	25 (50.0%)	25 (50.0%)	
Surgical	50 (50.0%)	25 (50.0%)	25 (50.0%)	

*SD* standard deviation

^*^Significant *p*-value

^**^Nutritional status was documented in only 68 medical reports

### Clinical history and physical examination

The average symptom duration showed no significant difference between the knee and hip groups (*p* = 0.158). Disparities, however, emerged in the frequency of start-up pain and pain during stair ascent (*p* = 0.003 and *p* = 0.047, respectively). The hip group reported start-up pain more frequently, while stair ascent pain was more prevalent among patients with gonarthrosis. Relating to other pain scenarios, pain management approaches and prior therapeutic actions, no marked discrepancies were noted between the two subgroups (*p* > 0.05) (Table 2).

**Table 2:** Clinical history: symptoms duration, pain manifestations and previous therapeutic interventions in knee and hip arthrosis groups

	Total (*n* = 100)	Knee (*n* = 50)	Hip (*n* = 50)	*p*-value
Complaints duration (years)				0.158
Range	0.04–14	0.04–14	0.08–10	
Mean (± SD)	2.68 (± 3.00)	3.11 (± 3.44)	2.26 (± 2.45)	
Resting pain				0.834
Yes	35 (35.0%)	17 (34.0%)	18 (36.0%)	
No	65 (65.0%)	33 (66.0%)	32 (64.0%)	
Night pain				0.685
Yes	42 (42.0%)	20 (40.0%)	22 (44.0%)	
No	58 (58.0%)	30 (60.0%)	28 (56.0%)	
Start-up pain				0.003*
Yes	53 (53.0%)	19 (38.0%)	34 (68.0%)	
No	47 (47.0%)	31 (62.0%)	16 (32.0%)	
Load pain				0.307
Yes	96 (96.0%)	49 (98.0%)	47 (94.0%)	
No	4 (4.0%)	1 (2.0%)	3 (6.0%)	
Climbing stair pain				0.047*
Yes	29 (29.0%)	19 (38.0%)	10 (20.0%)	
No	71 (71.0%)	31 (62.0%)	40 (80.0%)	
Walking distance				0.216
Reduced	62 (62.0%)	28 (56.0%)	34 (68.0%)	
Not reduced	38 (38.0%)	22 (44.0%)	16 (32.0%)	
Reduced life quality				0.841
Reduced	49 (49.0%)	25 (50.0%)	24 (48.0%)	
Not reduced	51 (51.0%)	25 (50.0%)	26 (52.0%)	
On-demand painkillers				0.130
Yes	31 (31.0%)	12 (24.0%)	19 (38.0%)	
No	69 (69.0%)	38 (76.0%)	21 (62.0%)	
Long-term painkillers				0.198
Yes	32 (32.0%)	19 (38.0%)	13 (26.0%)	
No	68 (68.0%)	31 (62.0%)	37 (74.0%)	
Physiotherapy				0.383
Yes	30 (30.0%)	13 (26.0%)	17 (34.0%)	
No	70 (70.0%)	37 (74.0%)	33 (66.0%)	
Intraarticular injection				0.373
Yes	28 (28.0%)	16 (32.0%)	12 (24.0%)	
No	72 (72.0%)	34 (68.0%)	38 (76.0%)	

*SD* standard deviation

^*^Significant *p*-value

Upon clinical examination, limited joint movement was found in 32% of the knee participants and 66% of those in the hip group (*p* < 0.001). A thorough breakdown of clinical examination outcomes can be referenced in Table 3a for the knee group and Table 3b for the hip group.

**Table 3:** Physical examination outcomes in knee (a) and hip (b) patient cohorts

a		b	
	Knee (*n* = 50)		Hip (*n* = 50)
Restricted joint mobility		Restricted joint mobility	
Yes	16 (32.0%)	Yes	33 (66.0%)
No	34 (68.0%)	No	17 (34.0%)
PF crepitation		Impingement pain	
Yes	16 (32.0%)	Yes	43 (68.0%)
No	34 (68.0%)	No	7 (14.0%)
Joint swelling		Axial compression pain	
Yes	9 (18.0%)	Yes	7 (14.0%)
No	41 (82.0%)	No	43 (86.0%)
Patella facet tenderness		Groin PP	
Yes	14 (28.0%)	Yes	27 (54.0%)
No	36 (72.0%)	No	23 (46.0%)
Clarke’s sign		Trochanteric PP	
Yes	29 (58.0%)	Yes	8 (16.0%)
No	21 (42.0%)	No	42 (84.0%)
Joint line PP		Motion pain	
Yes	31 (62.0%)	Yes	23 (46.0%)
No	19 (38.0%)	No	27 (54.0%)
Meniscus test			
Reduced	10 (20.0%)		
Not reduced	40 (80.0%)		
Mediolateral laxity			
Yes	12 (24.0%)		
No	38 (76.0%)		
Leg axis alignment			
Neutral	27 (54.0%)		
Varus	13 (26.0%)		
Valgus	10 (20.0%)		

*PF* patellofemoral, *PP* palpation pain

Radiological evaluations showed varying osteoarthritis severities between the knee and hip subsets. A distinct difference in KL scores was observed (*p* = 0.004). Notably, grade 4 osteoarthritis had a higher prevalence in the hip group (42%) compared with the knee group (10%). Despite the limited availability of MRI data (17% of patients), degenerative changes were consistently observed in all assessed cases (Table 4).

**Table 4:** Radiological findings and osteoarthritis severity based on the Kellgren-Lawrence classification in knee and hip disorders

	Total (*n* = 100)	Knee (*n* = 50)	Hip (*n* = 50)	*p*-value
KL score				0.004*
Grade 1**	10 (10.0%)	6 (12.0%)	4 (8.0%)	
Grade 2	20 (20.0%)	13 (26.0%)	7 (14.0%)	
Grade 3	44 (44.0%)	26 (52.0%)	18 (36.0%)	
Grade 4	26 (26.0%)	5 (10.0%)	21 (42.0%)	
Degeneration signs in MRI				0.183
Yes	17 (17.0%)	11 (22.0%)	6 (12.0%)	
No	0 (0.0%)	0 (0.0%)	0 (0.0%)	
N/A	83 (83.0%)	39 (78.0%)	44 (88.0%)	

*KL* Kellgren and Lawrence, *N/A* not available

^*^Significant *p*-value

^**^Grade 0 was not considered, as it was not present in any clinical record

### Patient characteristics across recommended treatments

The average age of patients recommended for surgical intervention was significantly higher than those advised for conservative treatment. While the duration of symptoms did not differ significantly between the two groups, there were notable differences in the occurrence of pain under various conditions, reduced walking distance, long-term painkiller use and reduced life quality. Notably, a higher proportion of patients in the surgical group had restricted joint mobility, higher KL scores and exhibited specific clinical signs such as Clarke’s sign and joint line palpation pain for the knee and impingement and groin palpation pain for the hip (Table 5).

**Table 5:** Comparison of clinical and radiological parameters in conservative versus surgical subgroups

	Conservative (*n* = 50)	Surgical (*n* = 50)	*p*-value
Age (years)			< 0.001*
Range	35–81	50–88	
Mean (± SD)	59.3 (± 10.5)	70.0 (± 9.03)	
Complaints duration (years)			0.266
Range	0.04–14	0.08–10	
Mean (± SD)	2.35 (± 2.99)	3.02 (± 3.01)	
Resting pain	11 (22.0%)	24 (48.0%)	0.006*
Night pain	15 (30.0%)	27 (54.0%)	0.015*
Start-up pain	24 (48.0%)	29 (58.0%)	0.316
Load pain	47 (94.0%)	49 (98.0%)	0.307
Reduced walking distance	21 (42.0%)	41 (82.0%)	< 0.001*
Long-term painkillers	7 (14.0%)	25 (50.0%)	< 0.001*
Reduced life quality	6 (12.0%)	43 (86.0%)	< 0.001*
Restricted joint mobility	12 (24.0%)	37 (74.0%)	< 0.001*
Clarke’s sign^1^	11 (22.0%)	18 (36.0%)	0.045*
Joint line palpation pain^1^	10 (20.0%)	21 (42.0%)	0.001*
Impingement pain^2^	19 (38.0%)	24 (48.0%)	0.042*
Groin palpation pain^2^	9 (18.0%)	19 (38.0%)	0.011*
KL score			< 0.001*
Grade 1	10 (20.0%)	0 (0.0%)	
Grade 2	15 (30.0%)	5 (10.0%)	
Grade 3	24 (48.0%)	20 (40.0%)	
Grade 4	1 (2.0%)	25 (50.0%)	

*SD* standard deviation, *KL* Kellgren and Lawrence

^*^Significant *p*-value

^1^refers exclusively to the knee subgroup

^2^refers exclusively to the hip subgroup

### ChatGPT-4 analysis

When assessing the diagnostic accuracy of ChatGPT-4 recognizing gonarthrosis and coxarthrosis, the concordance with a physician’s diagnosis was 100% for the total cases.

In examining the alignment of therapeutic recommendations between ChatGPT-4 and the orthopaedic specialists, there was an observed concordance in therapeutic recommendations in 83% of the total cases examined.

In cases of gonarthrosis, the concordance rate stood at 82% (41 out of 50 cases), and similarly, for coxarthrosis, the concordance was marginally higher, with an 84% match (42 out of 50 cases) between the model’s suggestions and the involved clinicians’ recommendations (Table 6).

**Table 6:** Concordance between ChatGPT-4 and orthopaedic specialist

	Total (*n* = 100)	Knee (*n* = 50)	Hip (*n* = 50)
Diagnosis			
Concordance	100 (100.0%)	50 (100.0%)	50 (100.0%)
No concordance	0 (0.0%)	0 (0.0%)	0 (0.0%)
Therapeutic recommendation			
Concordance	83 (83.0%)	41 (82.0%)	42 (84.0%)
No concordance	17 (17.0%)	9 (14.0%)	8 (16.0%)

A significant difference was found in the age of the patients recommended for operative treatment by ChatGPT-4 compared with those recommended for conservative treatment (*p* = 0.002), with the operative group being older on average.

Using the KL osteoarthritis grading as a reference, and as a control mechanism for the plausibility of the decisions made, we analysed ChatGPT-4’s surgical therapy recommendations for patients with mild-to-moderate (grades 1–2) and severe (grades 3–4) arthrosis. Our analysis revealed a significant association (*p* < 0.001) between arthrosis severity and ChatGPT-4’s therapeutic recommendations.

**Fig. 2:**
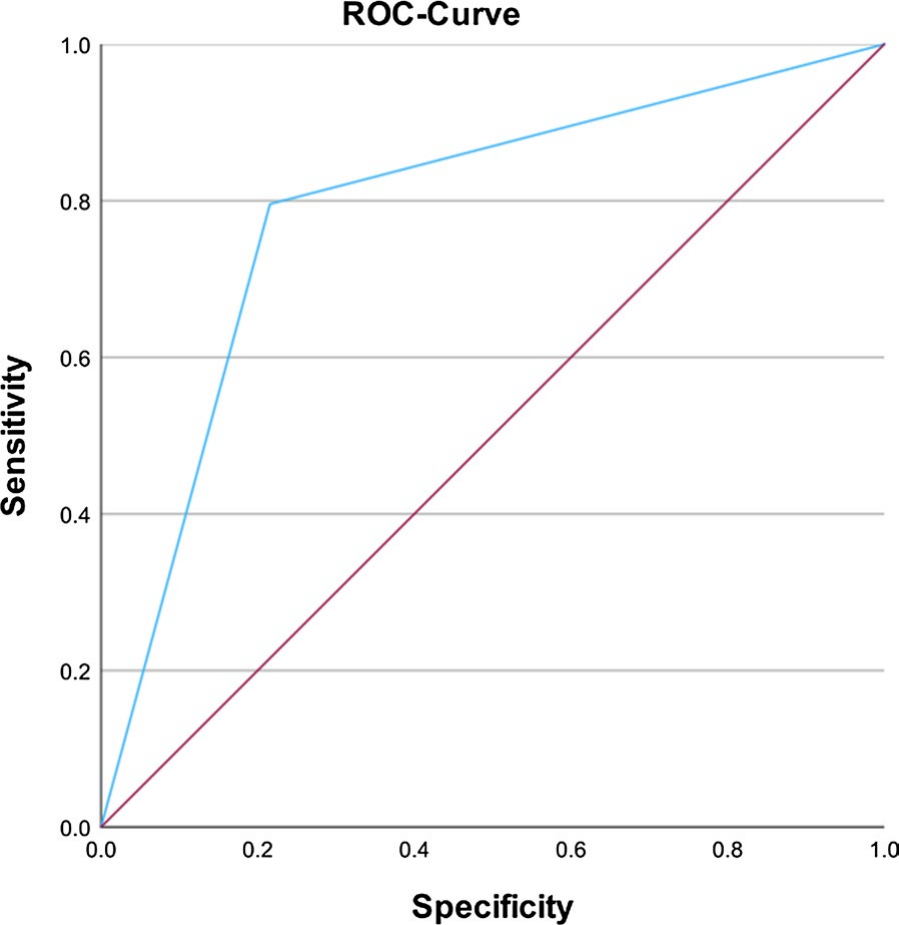
ROC curve for therapeutic advice by ChatGPT for operative treatment. The area under the curve (AUC) is 0.79

In the assessment of ChatGPT-4’s therapeutic recommendation capabilities based on the clinical records, ChatGPT-4 displayed a sensitivity of 78% for operative therapy recommendations, reflecting its ability to recommend operative measures that aligned with the suggestions of orthopaedic specialists. The specificity for operative recommendations, which refers to the accuracy in identifying cases suitable for conservative treatment, was assessed to be 80% (Fig. 2). The associated positive predictive value (PPV) and negative predictive value (NPV) for the operative therapy recommendations stood at 79.6% and 78.4%, respectively.

An interrater reliability analysis was conducted to determine the consistency of therapeutic recommendations between ChatGPT-4 and the orthopaedic specialists. The Cohen’s Kappa coefficient was found to be 0.580 (*p* < 0.001). This coefficient suggests a moderate-to-good level of agreement between the raters [15].

Logistic regression analysis, specifically the stepwise forward selection method, was employed to assess the influence of several variables on the therapeutic recommendation of ChatGPT, including pain at rest, pain during the night, start-up pain, reduced walking distance, reduced quality of life, continuous pain medication and pain during movement.

Multivariable logistic regression demonstrated that reduced quality of life [odds ratio (OR) 49.97, confidence interval (CI) 5.69–439.13.6, *p* < 0.001], as well start-up pain (OR 12.54, CI 1.31–120.0, *p* = 0.028) have a significant impact on the recommendation for surgery by ChatGPT-4.

## Discussion

This study aimed to evaluate the capability of ChatGPT-4 in diagnosing and advising treatments for real patient cases of gonarthrosis and coxarthrosis, comparing its outcomes directly with those of orthopaedic specialists.

ChatGPT-4 aligned with physicians in its diagnostic capability for arthrosis, emphasizing its potential for simple diagnostic tasks. However, reference to the work of Rao et al. [16] is enlightening. They found that while ChatGPT achieved an accuracy rate of 71.7%, it lagged in differential diagnosis and clinical management relative to broader medical questions. This underscores the importance of viewing AI tools such as ChatGPT-4 as supplementary aids, rather than replacements, for medical judgment.

Our findings also highlighted a considerable correspondence between ChatGPT-4’s therapeutic advice and specialist recommendations, evidenced by an 83% agreement rate in the reviewed cases. This is consistent with results from Harskam et al. [17], where ChatGPT’s recommendations matched actual medical advice in 90% of simulated cardiac cases. However, they also noted that ChatGPT occasionally lacked comprehensive or appropriate advice for more complex cases, especially when compared with expert feedback. Similarly, Nastasi et al. [18] observed that while 93% of ChatGPT’s responses to advice-seeking vignettes were in line with clinical guidelines, the system did not always provide specific medical advice, occasionally offering generalized or no advice at all. Furthermore, Rajjoub et al. [19], who assessed and compared ChatGPT’s responses with clinical questions and recommendations set forth by the 2011 North American Spine Society (NASS) Clinical Guideline for the Diagnosis and Treatment of Degenerative Lumbar Spinal Stenosis (LSS), found that ChatGPT’s responses integrated findings in the contemporary literature on LSS. This suggests the potential of incorporating ChatGPT into the spine surgeon’s workflow to aid the decision-making process for LSS diagnosis and treatment.

Despite these promising outcomes, it is important to acknowledge the limitations of ChatGPT-4 as a stand-alone solution. For example, our results showed that while the algorithm exhibits significant diagnostic competence, it does not fully replicate the expertise of an experienced orthopaedic surgeon. Specifically, the sensitivity and specificity of ChatGPT-4 in advising operative therapy were 78% and 80%, respectively, and the Cohen’s Kappa coefficient indicated only moderate agreement.

For instance, Kaarre et al. [20] investigated the use of LLMs by presenting queries related to anterior cruciate ligament (ACL) surgery to ChatGPT and found that it exhibited fair accuracy in generating correct responses in approximately 65% of the presented clinical cases. Although ChatGPT showed potential as a supplementary tool for acquiring orthopaedic knowledge and was able to effectively adapt to diverse target audiences, it could not replace the expertise of orthopaedic sports medicine surgeons in diagnostic and treatment planning endeavours due to its limited understanding of the orthopaedic branch and potential for erroneous responses.

Still, the substantial alignment observed in our study suggests that ChatGPT-4 can accurately interpret and utilize clinical history, physical examination and radiological assessment to make recommendations that reflect real-world clinical decisions.

To our knowledge, this is the first study to evaluate similar capabilities of ChatGPT-4 in the clinical practice of arthroplasty surgery. While there have been previous studies suggesting the potential utilization of AI systems in orthopaedics, such as a review by Cheng et al. [11] that outlined possible roles of GPT-4 across various stages of arthroplasty care, none have actually investigated these features in a clinical setting. Hence, direct comparison with other studies is challenging. Still, our results align with the emerging body of research that underscores the potential benefits of AI in healthcare settings [21–23].

This study, however, is not without limitations. A key limitation of this study is the precision of ChatGPT-4’s diagnostic and treatment recommendations. Despite substantial agreement with specialists’ diagnoses and therapeutic suggestions, the AI’s sensitivity and specificity in proposing operative interventions, and its moderate Cohen’s Kappa coefficient, did not achieve the consistent accuracy typical of seasoned orthopaedic surgeons. This difference highlights the intricate nature of clinical decision-making, a domain where AI still cannot replicate the profound expertise inherent to human judgment.

Another concern is the relatively small size of the patient population, which might restrict the broader application of our findings. While this is understandable for an exploratory study, it remains essential to carry out larger-scale investigations to validate these results. A significant limitation of ChatGPT-4 in our study is its inability to account for individual patient preferences, crucial when suggesting optimal suited treatments. The completeness and accuracy of patients’ medical histories present another challenge. Some critical information might have been conveyed verbally and not documented. Moreover, since physicians composed the medical records we employed, it is possible that the data was structured in a way that favoured ChatGPT-4’s interpretation. Our research did not investigate scenarios in which patients interacted directly with ChatGPT-4 without a physician intermediary. However, a study by Mika et al. [24] examined ChatGPT’s proficiency in answering patients’ common questions about total hip arthroplasty. Although some answers needed further elaboration, the Chabot provided generally unbiased and evidence-based responses, even for controversial issues. Given these findings, ChatGPT shows promise as a useful tool for patients before orthopaedic consultations. ChatGPT-4, while proficient in data interpretation and pattern recognition, still cannot emulate the intricate expertise and nuanced clinical judgment of experienced orthopaedic surgeons, who draw on years of direct patient care, hands-on surgical experience, and intuitive understanding of individual patient needs beyond what can be quantified in data alone.

## Conclusion

ChatGPT-4 consistently matched physicians’ diagnoses of gonarthrosis and coxarthrosis, demonstrating a 100% diagnostic agreement. Furthermore, the therapeutic recommendations provided by ChatGPT-4 aligned with real-world clinical practices, with an 83% agreement rate. In suggesting surgical treatments, the system had a sensitivity of 78% and a specificity of 80%. It also exhibited moderate consistency with orthopaedics specialists, as evidenced by a Cohen’s Kappa coefficient of 0.580 (*p* < 0.001). A multivariable logistic regression indicated that ChatGPT-4’s surgical recommendations were associated with decreased quality of life (OR 49.97, *p* < 0.001) and start-up pain (OR 12.54, *p* = 0.028). While these results are encouraging, one must consider the intricate knowledge and experience of orthopaedic surgeons, which may not be entirely replicated by AI systems. Continued research is crucial to understand the full extent of AI’s potential in diagnosing various conditions in larger patient groups and to determine the most suitable clinical settings for its application.

### Supplementary Information

**Additional file 1.** Individual clinical query input to the ChatGPT-4 case prompt and the respective caserelated ChatGPT-4 response.

## Data Availability

All data generated or analysed during this study are included in this published article and its supplementary information files.
