# Supplementary material for: Arthrosis diagnosis and treatment recommendations in clinical practice: an exploratory investigation with the generative AI model GPT-4
Source: J Orthop Traumatol. 2023 Nov 28;24:61. doi: 10.1186/s10195-023-00740-4 (PMC10684473; doi:10.1186/s10195-023-00740-4)
Supplement: Supplementary file 1 — Additional file 1. Individual clinical query input to the ChatGPT-4 case prompt and the respective caserelated ChatGPT-4 response. [file 10195_2023_740_MOESM1_ESM.docx]

1. **Clinical Query Input to ChatGPT-4 - Case Prompt**

*“Hi, I'm an orthopedic physician, and I am conducting a clinical study to test the capabilities of ChatGPT-4 (you) in providing a diagnosis and therapeutic recommendations. The decisions you make are based only on the anonymized text of discharge letters from a series of outpatient clinic visits. I will send you anamnesis with a physical examination and radiographic image description (the texts are in German, but you will still answer in English). You will suggest a diagnosis, possibly with a differential diagnosis, and therapeutic recommendations.*

*Prioritize your decisions and express them as a percentage based on the importance/order you assign. Be concise (no explanations or repetitions of provided prompt); simply provide a specific but detailed diagnosis. I am aware that you can't provide medical information, so please refrain from giving disclaimers of any kind.”*

To ensure that ChatGPT-4 processed the data as an orthopedic physician would, it was provided with specific instructions. The model was tasked with identifying a primary diagnosis and, if applicable, generating a differential diagnosis in order of probability.

ChatGPT-4 was also asked to provide therapeutic advice. By doing so, the study could assess not just the diagnostic capabilities of the AI but its therapeutic reasoning as well.

The choice to instruct the model to be concise and avoid redundant information was indented to minimize any confounder. Additionally, the direction to avoid disclaimers was a conscious decision to ensure that the AI's outputs were purely based on its data-driven insights, rather than potential legal or ethical considerations.

The structure and specifics of the prompt were designed to examine whether such technology could potentially parallel, complement, or even enhance the diagnostic and therapeutic processes of human orthopedic physicians.

1. **Case-Based ChatGPT-4 Response**

***DIAGNOSIS:***

- *Initial Coxarthrosis (hip osteoarthritis) of the left hip - 70%*
- *Hip dysplasia - 30%*

***THERAPEUTIC RECOMMENDATIONS:***

- *Continue non-surgical management - analgesics as needed, physiotherapy, and weight management if appropriate - 60%*
- *Re-evaluate efficacy of Hyaluronic acid injections, consider discontinuation if no significant relief - 20%*
- *If non-surgical management fails, consider total hip arthroplasty (hip replacement surgery) - 20%.*

To further illustrate ChatGPT-4's diagnostic and therapeutic approach, here's a selected example from its generated responses. This response is presented as an exemplar, intended to offer a snapshot into the model's medical decision-making process based on input clinical data.

Upon analyzing the patient's provided medical history and physical examination results, ChatGPT-4 rendered a diagnosis. Predominantly, it identified "Initial Coxarthrosis (hip osteoarthritis) of the left hip" with a prominence of 70%. Concurrently, "Hip dysplasia" was also discerned as a potential concern, attributed a 30% weightage.

From a therapeutic perspective, ChatGPT-4 suggested a series of interventions. The primary emphasis was on the continuation of non-surgical management techniques, including the use of analgesics, physiotherapy, and potential weight management. Furthermore, the model advised assessing the efficacy of Hyaluronic acid injections, underlining reconsideration if they failed to provide notable relief. If conservative measures were unsuccessful, the prospect of total hip arthroplasty was presented as a viable consideration.

ChatGPT-4's decision weightings, expressed in percentages, represent the model's estimation of relevance or importance based on its training data and recognized patterns within the provided information.
